# Renshen Baidu Powder Attenuated Intestinal Inflammation and Apoptosis in Ulcerative Colitis Rats through the Inhibition of PI3K/AKT/NF-*κ*B Signaling Pathway

**DOI:** 10.1155/2022/5234025

**Published:** 2022-07-30

**Authors:** Peixu Zhang, Xiaobo Zhang, Peiyu Xiong, Chun Zhong, Zhen Zhou, Bo Jia, Xinglong Liu

**Affiliations:** ^1^School of Basic Medicine, Chengdu University of Traditional Chinese Medicine, Chengdu 610072, China; ^2^Sichuan Second Hospital of Traditional Chinese Medicine, Chengdu 610014, China; ^3^Menzies Institute for Medical Research, University of Tasmania, Hobart TAS7000, Australia

## Abstract

**Objective:**

Renshen Baidu Powder (RBP) is a famous classic compound of traditional Chinese medicine (TCM) and is commonly used for treating ulcerative colitis (UC). However, the pharmacological mechanism of RBP in treating UC remains unclear. This study investigates the possible mechanism of RBP for UC treatment by network pharmacological analysis and rat validation.

**Methods:**

First, the main chemical constituents of RBP were identified using ultrahigh-performance liquid chromatography quadrupole Orbitrap high-resolution mass spectrometry (UHPLC-Q-Orbitrap-HRMS). Then, we obtained targets of identified compounds from the SwissTargetPrediction database and targets associated with UC from GeneCards database. Furthermore, Gene Ontology (GO) and Kyoto Encyclopedia of Genes and Genomes (KEGG) were used to analyze the metabolism-related signaling pathways affected by RBP. Hematoxylin-eosin (HE) staining was used to observe the pathological change of colon in UC rats after treating RBP, and terminal deoxynucleotidyl-transferase (TdT)-mediated dUTP Nick end labeling (TUNEL) staining was used to detect apoptosis after RBP treatment. The enzyme-linked immunosorbent assay (ELISA) was employed to evaluate cytokine levels of TNF-*α*, IL-1*β*, and IL-6. The protein expressions of Bax, Bcl-2, PI3K, AKT, and NF-*κ*B in colonic tissue were detected using immunohistochemistry (IHC). Real-time quantitative polymerase chain reaction (RT-QPCR) was employed to evaluate mRNA expression of PI3K, AKT, and NF-*κ*B.

**Results:**

We found a total of 24 main compounds and 329 potential targets related to UC. According to KEGG results, 3 main pathways were identified as responsible for UC, including PI3K-AKT, HIF-1, and VEGF signaling pathway. Animal experiments showed that RBP treatment significantly attenuated colon damage in rats with UC. Mechanistically, RBP could inhibit PI3K/AKT/NF-*κ*B pathway; decrease cell apoptosis; and downregulate the expression of TNF-*α*, IL-1*β*, and IL-6.

**Conclusions:**

This study demonstrated that RBP may exert anti-inflammatory and antiapoptotic therapeutic benefits in UC by regulating the PI3K/AKT/NF-*κ*B signaling pathways, providing a scientific basis for understanding the mechanism of RBP against UC.

## 1. Introduction

Ulcerative colitis (UC) is a chronic nonspecific inflammatory disease and a subtype of inflammatory bowel disease (IBD). The incidence of UC continues to increase, and nearly 30% of cases can develop into colon cancer, with a mortality rate greater than 50% [1, 2]. The etiology and pathogenesis of UC are complex. Previous research suggested that it may be related to the abnormal immune regulation of intestinal wall mucosa, mucosal barrier defects, persistent intestinal infections, and the mutual involvement of genetic and environmental factors [3]. Among the genetically susceptible individuals, as a joint result of exposure to the environmental factors (e.g., regional differences and dietary structure) and altered intestinal flora, the intestinal epithelial barrier can be destroyed, along with the increased mucosal permeability and long-term exposure to a large number of antigens by intestinal tissues. This can lead to the overreaction or misidentification by the intestinal immune system, the activation of macrophages and lymphocytes, the infiltration of inflammatory cells into the intestinal mucosa, and the release of a large number of pro-inflammatory factors (e.g., tumor necrosis factor-*α* [TNF-*α*]; transforming growth factor-*β* [TGF-*β*]; and interleukin family [ILs]—pro-inflammatory factors interleukin-1 beta [IL-1*β*], interleukin-2 [IL-2], interleukin-6 [IL-6], and interleukin-8 [IL-8]) and some inflammatory mediators such as myeloperoxidase (MPO), histamine and 5-hydroxytryptamine, reactive oxygen species (ROS) and reactive nitrogen species (RNS), prostaglandins (PGs), and arachidonic acid (ARA), following the activation of body's immune system. The inflammatory response is amplified step by step, eventually leading to the tissue damage and a series of pathological manifestations under the eyes or microscope [4]. These factors do not act independently but interact with each other during the whole process leading to the development of ulcerative colitis. Among them, the enhancement of inflammatory stimulating factors and the weakening of immune regulation play key roles [5, 6].

Herbal medicine or phytotherapy is a form of treatment in complementary medicine that has been practiced for centuries, especially in the field of traditional Chinese medicine (TCM) [7]. Complementary and alternative medicine has been reported to be used by 30% to 50% of patients with IBD [8]. Herbal medicines have become an attractive alternative to alleviate UC [9, 10]. Renshen Baidu Powder (RBP) is a famous classic formula consisting of twelve medicinal herbs: Chai Hu (*Bupleurum chinense* DC.), Chuan Xiong (*Ligusticum Chuanxiong* Hort.), Ren Shen (*Panax ginseng* C.A.Mey.), Qian Hu (*Angelica decursiva* (Miq.) Franch. & Sav.), Gan Cao (*Glycyrrhiza glabra* L.), Jie Geng (*Platycodon grandiflorus* (Jacq.) A.DC.), Qiang Huo (*Notopterygium incisum* K.C.Ting ex H.T.Chang), Du Huo (*Angelica dahurica* (Hoffm.) Benth. & Hook.f. ex Franch. & Sav.), Fu Ling (*Smilax glabra* Roxb.), Zhi Ke (*Citrus × aurantium* L.), Bo He (*Mentha canadensis* L.), and Sheng Jiang (*Zingiber officinale* Roscoe), which has been used to treat abdominal pain, diarrhea, and mucus-purulent-blood stools in China for thousands of years.

In modern clinical practice, RBP is often used to treat UC [11, 12], diarrheal irritable bowel syndrome [13], and other diseases with abdominal pain and diarrhea as the main clinical manifestation, with a favorable therapeutic efficacy. Jiyong Li used RBP to treat 49 cases of UC patients and counted the efficacy after 4 weeks, the total effective rate of the treatment group was 89.8% [14]. In the experimental study, Peiyu Xiong et al. observed the effect of RBP on the intestinal mucosal barrier of 2,4,6-trinitro-benzenesulfonicacid (TNBS)-induced UC rats and its possible mechanism. They found that RBP could effectively improve the symptoms of UC rats and its mechanism might be related to the inhibition of inflammatory, promoting intestinal epithelial tight junction repair and improving intestinal mucosal permeability [15]. Previous studies have also shown that RBP can reduce gastric residual rate, increase small intestine propulsion rate, promote recovery of gastrointestinal function, and increase junctional adhesion molecule protein expression, thereby regulating intestinal epithelial cell tight junctions and improving intestinal mucosal damage in UC rats, which may be one of the mechanisms of RBP in the treatment of UC [16]. Herbs in the composition of RBP have pleiotropic pharmacological effects such as anti-inflammation, promotion of cell proliferation, inhibition of apoptosis, antioxidation, and antitumor [17–24]. Although the therapeutic effects of RBP and its constituent herbs on UC have been demonstrated in clinical and experimental studies, the complex interactions between active compounds and target proteins remain unclear. Besides, the signaling pathways involved are not completely understood.

Network pharmacology is an emerging approach based on the theory of systems biology, believing life is a complex system and diseases that cause changes in body mechanisms and functions are caused by multiple genes and protein interactions and changes [25]. The prediction of drug efficacy through drug structural and functional similarity and synthesis of multiple action relationships between biological targets and biological effector molecules, is in line with the characteristics of multi-component, multi-target, and multi-pathway of Chinese herbal medicine and is an effective approach to predict the mechanism of action and effect of Chinese medicine compound [26].

In this study, we investigated the interaction between RBP compounds and potential targets of UC. Through network pharmacology and experimental validation, we aimed to provide new evidence to support the pharmacological role and potential mechanisms of RBP in treating UC.

## 2. Materials and Methods

### 2.1. Materials and Reagents

The RBP herbs were purchased from Chengdu Tong Ren Tang Co., Ltd. and were identified as authentic by the Teaching and Research Department of Chinese Medicine Identification of Chengdu University of Traditional Chinese Medicine (the constituent herbs of the formulation and full botanical plant names are listed in Supplementary File 1). Sulfasalazine enteric-coated tablets (SASP) are manufactured by Shanghai Xinyi Tianping Pharmaceutical Co., Ltd. The manufacturers and product numbers of the reagents used in this study are described in Supplementary File 2.

### 2.2. Preparation of RBP and UHPLC-Q-Orbitrap-HRMS

The 12 herbs were mixed and boiled twice with 8 times water (volume/weight) for 30 min each, filtered, and concentrated. 200 *μ*L RBP solution was dissolved in 1 mL methanol and vortexed for 10 min. Then the mixture was centrifuged at 20000 × *G* for 10 min and filtered through a 0.22-*μ*m microporous membrane. The main ingredients of RBP were measured by ultrahigh-performance liquid chromatography quadrupole Orbitrap high-resolution mass spectrometry (UHPLC-Q-Orbitrap-HRMS) (UltiMate 3000 RS, Thermo Fisher Scientific, USA). The precise details are given in Supplementary File 3.

### 2.3. Prediction of Potential Targets of RBP and Collection of Therapeutic Targets for UC

Potential targets of compounds were identified by UHPLC-Q-Orbitrap-HRMS. The simplified molecular-input line-entry specification (SMILES) structure of each component was uploaded on the SwissTargetPrediction database for target fishing. Targets associated with UC were collected from GeneCards (https://www.genecards.org). We used “ulcerative colitis” as index words and limited the species to “*Homo sapiens.*”

### 2.4. Intersection of UC and RBP Target Genes and Network Construction

Venn analysis was used to obtain the intersection of RBP and UC-related targets, the diagram was generated by https://www.bioinformatics.com.cn, an online platform for data analysis and visualization [27]. Afterwards, the “drug-component-target-disease” network was established by Cytoscape 3.7.1, a software used for evaluating the multi-target and multi-function therapeutic feature of RBP for UC.

### 2.5. Gene Ontology (GO) and Kyoto Encyclopedia of Genes and Genomes (KEGG) Pathway Enrichment Analyses

To investigate the bioinformatic annotation of common targets between RBP and UC, we used Bioconductor, a data package in *R* software (version 3.6.1) to run GO and KEGG enrichment analysis. The results were plotted by https://www.bioinformatics.com.cn. GO terms and KEGG pathways with a *p* value < 0.05 was considered statistically significant, and the top 20 GO enrichments and KEGG pathways with the highest counts were analyzed.

### 2.6. Animals

Male Sprague-Dawley (SD) rats (200 ± 20 g, 6–8 weeks) were purchased from Chengdu Da Shuo Biological Technology Co., Ltd (Chengdu, China). All rats were housed in the specific-pathogen-free (SPF) grade animal room of the Chengdu University of Traditional Chinese Medicine in a standard 12-h light/dark cycle with free access to water and food. The room temperature was maintained at 22 ± 2°C. The animal study was reviewed and approved by the Chengdu University of Traditional Chinese Medicine Experimental Animal Ethics Committee (no. 2020-21).

### 2.7. Experimental UC Rat Model and Administration

The replication of the UC model in rats was induced by TNBS/ethanol mixture [28]. After 3 days of adapting to the experimental environment, the rats were randomly divided into the non-UC rat group (*n* = 10) and the UC rat group (*n* = 50). The UC rat group was given a TNBS/ethanol mixture (100 mg/kg) enema. The non-UC rat group was given the corresponding volume of saline enema. After 7 days, 40 successful UC rats were randomly divided into 5 groups including the model group (*n* = 8), SASP group (0.3125 g/kg, *n* = 8), and three RBP groups (RBP-H, 31.2 g/kg, *n* = 8, RBP-M, 15.6 g/kg, *n* = 8, or RBP-L, 7.8 g/kg, *n* = 8). The RBP and SASP groups (note: SASP was used as a comparator to demonstrate the validity of the model in this study) were administered with the corresponding drugs, and the control and model groups were administered with an equal volume of saline until 14 days.

### 2.8. Histopathological Examination

At the end of the experiment, the colorectal length was measured after all rats were euthanized with pentobarbital anesthesia. Colon samples were collected and fixed in a 4% paraformaldehyde solution. Then, the tissue was embedded in paraffin to make paraffin sections and stained with hematoxylin and eosin, while a portion of colon tissue was frozen in liquid nitrogen. The stained sections of the colon were observed under a microscope (Nikon Eclipse E100, Tokyo, Japan) at 200× magnification.

### 2.9. Terminal Deoxynucleotidyl-Transferase (TdT)-Mediated dUTP Nick End Labeling (TUNEL) Assay

TUNEL was used to detect the apoptosis of intestinal epithelial cells. TUNEL staining was processed by an apoptosis two-step assay kit. The slides of the TUNEL stain were sealed by an anti-fluorescence quenching sealer. The images of the slides above were collected by NanoZoomer-S60 (Hamamatsu Photonics (China) Co., Ltd.) and analyzed by ImageJ.

### 2.10. Enzyme-Linked Immunosorbent Assay (ELISA)

Colon was collected and stored at −80°C. Subsequently, we took the colon milling with phosphate-buffered saline (PBS) to produce tissue homogenate. Protein content in colonic tissue homogenates was determined using the bicinchoninic acid (BCA) protein concentration determination kit. The concentration of IL-1*β*, IL-6, and TNF-*α* was determined using an ELISA kit.

### 2.11. Real-Time Quantitative Polymerase Chain Reaction (RT-QPCR)

Total RNA from the colons was isolated with Animal Total RNA Isolation Kit, and reverse transcription was performed using the 5× All-In-One MasterMix (with AccuRT Genomic DNA Removal kit) according to the manufacturer's instructions. Quantitative PCR analyses were performed on Bio‐Rad CFX96 System using the EvaGreen Express 2 × qPCR MasterMix-No Dye. Primers were designed and synthesized by Sangon Biotech (Shanghai) Co., Ltd. Polymerase chain reaction primers' gene sequences are described in Table 1.

### 2.12. Immunohistochemistry Analysis (IHC)

Colonic tissues from each group of rats were collected to make paraffin sections. These sections were mounted on positively charged adhesive glass slides (80312–3161, Citotest Labware Manufacturing Co., Ltd, Jiangsu, China) for IHC. Paraffin sections of colonic tissue were repaired with citrate antigen repair buffer and then blocked with 3% bovine serum albumin (BSA) at room temperature for 30 minutes. Tissues were then incubated with primary antibody diluted in PBS overnight at 4°C. Next, the sections were washed with PBS three times, and the tissues were incubated with secondary antibody diluted in PBS for 50 minutes at room temperature. Finally, the image acquisition of diaminobenzidine (DAB)-stained sections was performed using a digital pathology scanner (HS6, Shanghai Sunny Hengping Scientific Instrument Co., Ltd., Shanghai, China).

### 2.13. Statistical Analysis

All analyses were performed using SPSS 23.0 statistical software. Continuous data are presented as mean ± S.E.M and were analyzed with an independent-samples *t*-test or one-way analysis of variance (ANOVA) according to the number of groups. All tests were two-sided, and *P* < 0.05 was considered statistically significant.

## 3. Results

### 3.1. Identification of the Main Chemical Compounds of RBP

**Figure 1:**
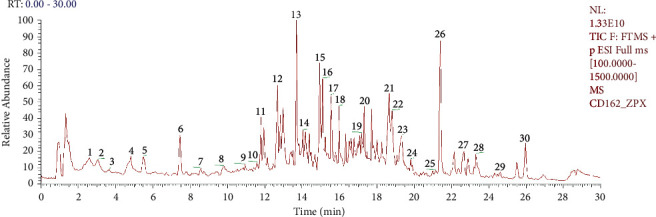
The total ion current (TIC) chromatogram of RBP in positive ion mode.

The main ingredients of RBP were analyzed by UHPLC-Q-Orbitrap-HRMS. The typical total ion chromatogram (TIC) of RBP on positive ion mode is shown in Figure 1. According to the reference standards, a total of 30 chemicals in positive ion were identified. Detailed information is described in Supplementary File 4.

### 3.2. Identification of Potential Targets for Compounds

We uploaded the corresponding canonical SMILES of the compounds in SwissTargetPrediction, and 606 therapeutic targets were obtained after eliminating the repetitive component targets. Using the search terms ‘ulcerative colitis', 4837 UC-related target genes were found in the GeneCards database.

### 3.3. Collection of Therapeutic Targets for RBP Acting on UC

**Figure 2:**
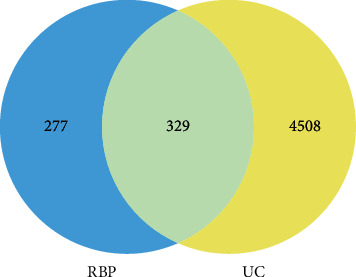
The 329 common genes between UC and RBP.

**Figure 3:**
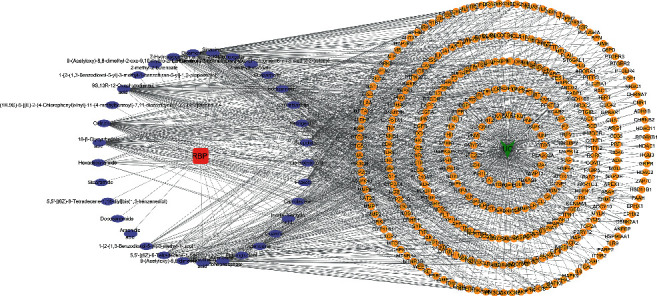
“Drug-compounds-targets-disease” network of RBP in UC. The red square represents the RBP drug, the purple oval nodes represent ingredients, the orange round nodes represent targets, and the green nodes represent the disease (UC).

After obtaining the RBP targets and the UC targets, we generated a Venn diagram by mapping the intersection (Figure 2). The intersection targets of compounds and disease were considered as the potential targets of RBP in the treatment of UC. The “drug-compounds-targets-disease” network was constructed by Cytoscape 3.7.1 (Figure 3). In the network, purple nodes represent compounds, orange nodes represent targets, and edges represent the association between one node and the other. Some compounds could interact with several targets related to UC.

### 3.4. GO and KEGG Enrichment

**Figure 4:**
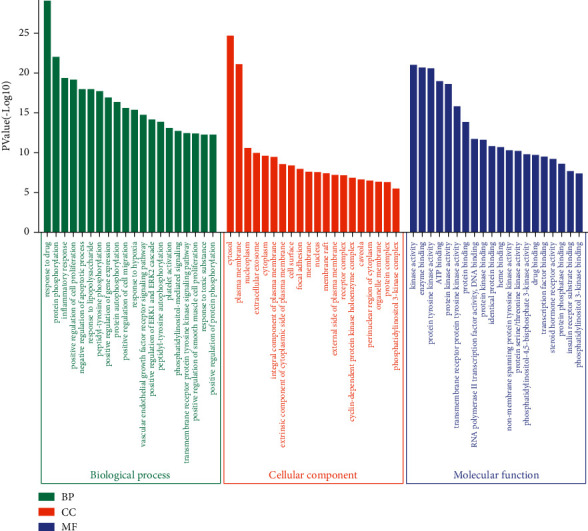
GO analysis of the 329 overlapping gene symbols associated with UC. The *X*-axis represents the categories in the GO of the target genes, while the *Y*-axis represents the *P* value (−log10) in the GO of the target genes.

**Figure 5:**
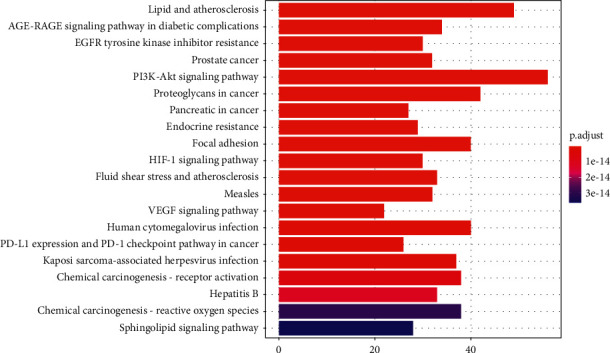
The KEGG pathway enrichment result of the 329 overlapping genes. The *X*-axis represents the fold enrichment of each pathway, the *Y*-axis represents the main pathways (*P* value <0.01), the size of the bar plot indicates target counts in each pathway, and the color of the bar plot indicates the *P* value.

**Figure 6:**
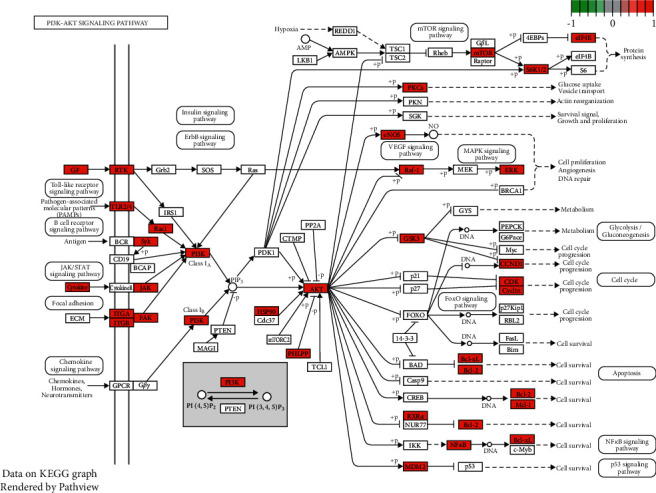
PI3K-AKT signaling pathway mapper. The arrows (⟶) represent the promoting effect and the T-arrows (⊣) represent the inhibiting effect. The enriched genes were marked as red while common genes of pathways were non-colored.

To reveal the potential pharmacological mechanisms of RBP in UC, we conducted GO and KEGG enrichment analyses. In the BP category of GO (Figure 4), the 329 intersected genes were significantly enriched in response to the drug, protein phosphorylation, inflammatory response, positive regulation of cell proliferation, negative regulation of apoptotic process, etc. KEGG enrichment analysis revealed that the 329 intersected genes were highly related to PI3K-AKT signaling pathway, HIF-1 signaling pathway, VEGF signaling pathway, etc (Figures 5 and 6).

### 3.5. Validation of the Efficacy of RBP in UC

**Figure 7:**
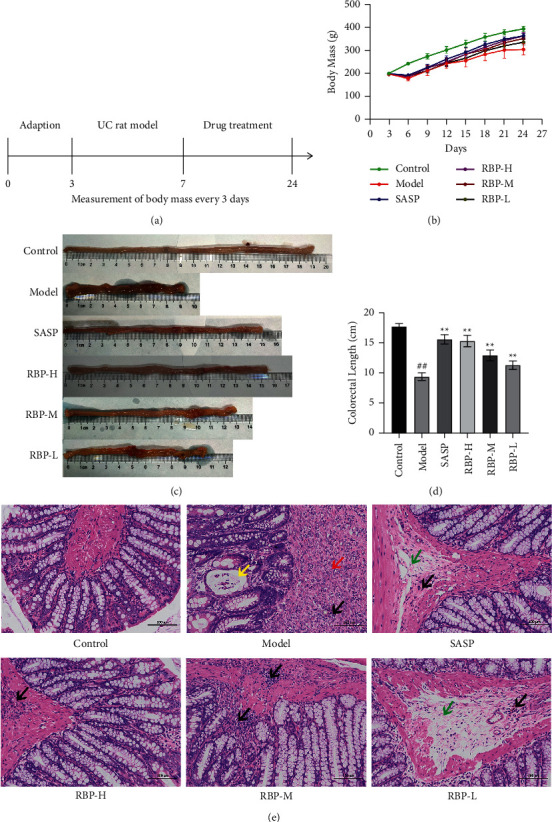
Assessment of the efficacy of RBP in UC. (a) Flow diagram of experiment arrangement. (b) The change of body mass in rats during the treatment (*n* = 6). (c) The morphological appearance of the colorectum. (d) The length of the colorectum (*n* = 6). (e) Typical histologically morphological changes in Control, Model, and treated UC rats. The red arrows represent neutrophil and macrophage infiltration, green arrows represent edema, yellow arrows represent residual heterosexual intestinal glands, and black arrows represent inflammatory cell infiltration. Compared with the control group: ^#^*P* < 0.05, ^##^*P* < 0.01; Compared with the model group: ^*∗*^*P* < 0.05, ^*∗∗*^*P* < 0.01. *n* = 6 for each group.

UC rats treated with RBP showed improvement in weight loss (Figure 7). To confirm the efficacy of RBP in UC, HE staining was used to reveal the histopathological changes in the colon. In the model group, we observed a large ulcerated area at the mucosal layer of the intestine, with the injury invading the muscular layer. The structure of the mucosal layer was basically lost at the ulcer foci, and the residual intestinal epithelial structures were visible on the surface. The ulcer was replaced by a mass of new proliferating connective tissue with extensive infiltration of neutrophils and macrophages. There was serious edema of local submucosa and infiltration of inflammatory cells. The SASP and RBP groups showed improved colonic mucosal lesions, in which the mucosa and submucosa began to show repair and healing, the symptoms of congestion and edema were reduced, the area of erosions and ulcers was reduced, and the infiltration of inflammatory cells in the surrounding tissues was decreased. The structure of the intestinal layers was clear, the mucosal epithelium was intact, the intestinal glands were abundant and neatly arranged, the cup-shaped cells in the intestinal gland epithelium were clearly visible, and the edema of local submucosa was mild with scattered infiltration of inflammatory cells. These results suggest that RBP could ameliorate colonic injury in the UC rat models (Figure 7).

### 3.6. Effect of RBP on Apoptosis of TUNEL in Intestinal Epithelial Cells

**Figure 8:**
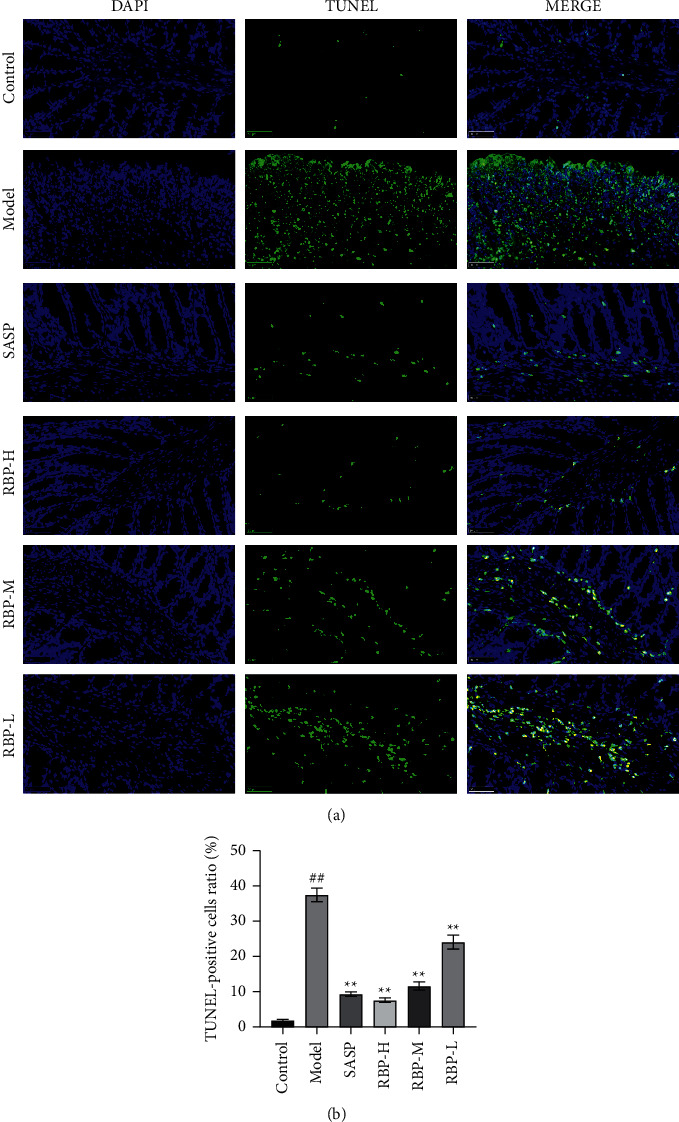
(a) Immunofluorescent staining of TUNEL (×400). Apoptotic cells were stained with green color, and the nucleus was stained with blue color. (b) Apoptosis rate of each group. Compared with the control group: ^#^*P* < 0.05, ^##^*P* < 0.01; Compared with the model group: ^*∗*^*P* < 0.05, ^*∗∗*^*P* < 0.01. *n* = 3 for each group.

To further investigate the protective effect of RBP on the intestinal mucosal barrier in UC rats, we assessed the apoptosis of intestinal epithelial cells by TUNEL. There were very few TUNEL-stained positive cells in the control group. Significant TUNEL-positive cells were observed in the sections of the model group, and the number of TUNEL-positive cells was significantly reduced after RBP and SASP treatment compared with the model group (Figure 8).

### 3.7. RBP Regulates Intestinal Inflammation and Apoptosis via PI3K/AKT/NF-*κ*B Pathway

**Figure 9:**
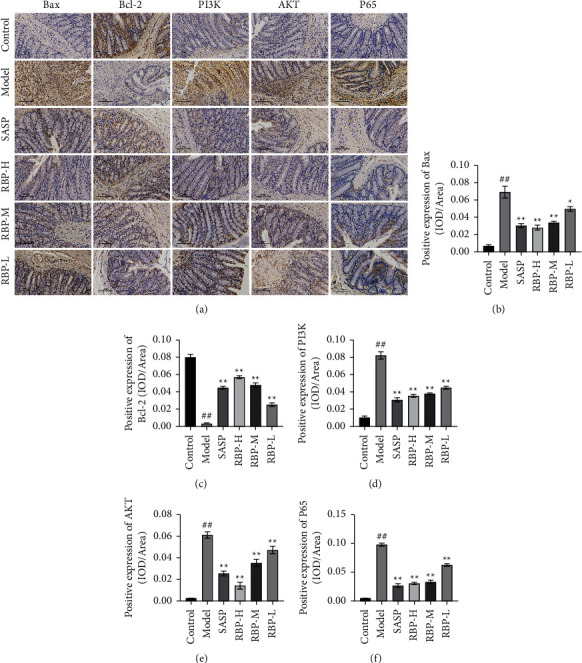
The effects of RBP on immunohistochemical staining of Bax, Bcl-2, PI3K, AKT, and NF-*κ*B p65 in the colonic tissue of UC rats (×200). (a) Representative immunohistochemical staining images of Bax, Bcl-2, PI3K, AKT, and NF-*κ*B p65. (b) Positive expression of Bax. (c) Positive expression of Bcl-2. (d) Positive expression of PI3K. (e) Positive expression of AKT. (f) Positive expression of NF-*κ*B p65. Compared with the control group: ^#^*P* < 0.05, ^##^*P* < 0.01; Compared with the model group: ^*∗*^*P* < 0.05, ^*∗∗*^*P* < 0.01. n = 5 for each group.

**Figure 10:**
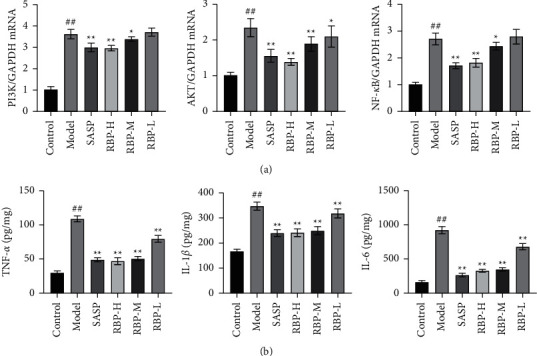
(a) Expression of PI3K, AKT, and NF-*κ*B mRNA. (b) The concentration of TNF-*α*, IL-6, and IL-1*β* in colonic tissue homogenate, respectively. Compared with the control group: ^#^*P* < 0.05, ^##^*P* < 0.01; Compared with the model group: ^*∗*^*P* < 0.05, ^*∗∗*^*P* < 0.01. *n* = 6 for each group.

To further investigate the underlying mechanism of action of RBP in the treatment of UC, we validated PI3K/AKT/NF-*κ*B pathway within the vivo experiment because this pathway is crucial in all related signaling pathways based on the analysis results' network pharmacology. The expression of PI3K/AKT/NF-*κ*B pathway-related proteins in rat colon tissue was examined using IHC. The results showed that the protein expression levels of B-cell lymphoma-2-associated *X* (Bax), PI3K, AKT, and NF-*κ*B were significantly higher in the model group compared with the control group. After treatments with RBP and SASP, the levels of these proteins were significantly downregulated. The expression of B-cell lymphoma-2 (Bcl-2) protein showed an opposite trend (Figure 9). Then, the expression of PI3K, AKT, and NF-*κ*B mRNA in the colon was examined, respectively. Compared to the control group, the mRNA expression levels of PI3K, AKT, and NF-*κ*B were considerably increased in the model group. However, the mRNA expression levels of PI3K, AKT, and NF-*κ*B were significantly downregulated after RBP and SASP treatments (Figure 10). These findings suggest that RBP can inhibit the PI3K/AKT/NF-*κ*B signaling pathway in UC rats. Furthermore, the concentration of TNF-*α*, IL-1*β*, and IL-6 in colon tissue homogenate was also measured, and our data showed that RBP could markedly reduce the levels of these biomarkers (Figure 10). These results indicated that RBP showed the anti-inflammatory and inhibitory effects on apoptosis through regulation of the PI3K/AKT/NF-*κ*B pathway.

## 4. Discussion

Ulcerative colitis is an inflammatory bowel disease (IBD) characterized by persistent diffuse inflammation of the rectum, colon, and ileum [29, 30]. The quality of life of UC patients has been seriously affected, which has imposed huge health and economic burdens on patients and become an important public health problem worldwide [31]. Clinical treatment goals include suppression of inflammation, promotion of mucosal healing, maintenance of symptomatic remission, reduction of recurrence, and prevention and control of complications. Currently, there are two kinds of treatments: drug treatment and surgical treatment, the mainstay of drugs used are corticosteroids, aminosalicylic acid, and immunomodulators [32, 33]. However, these treatments not only have potential risks of side effects such as infection and affecting the lymphatic immune system, but also may lead to recurrent UC attacks [34]. Therefore, the identification of effective and safe therapeutic drugs is of great importance.

Traditional Chinese formulas are composed of multiple herbs acting on complex diseases through multiple targets, pathways, and biological processes, and this feature could be beneficial to treating UC. In this study, the results of network pharmacology analysis showed that 24 compounds and 329 potential targets were related to UC. GO enrichment analysis revealed that the common targets of RBP and UC were significantly enriched in response to the drug, protein phosphorylation, inflammatory response, positive regulation of cell proliferation, negative regulation of apoptotic process, etc. In addition, pathway enrichment analysis identified 3 main pathways that were responsible for RBP in the treatment of UC, including PI3K-AKT signaling pathway, HIF-1 signaling pathway, and VEGF signaling pathway.

It is clear that UC is a complex polygenic, multifactorial disease and that these mutated genes influence the development of the disease through specific signaling pathways. Aberrant signaling pathways can influence the inflammatory process by dysregulating inflammatory response, thus playing a crucial role in the pathogenesis of UC. Phosphatidylinositol-3-kinase (PI3K) is a lipid second messenger associated with intracellular signaling, and the PI3K pathway is an important signaling pathway regulated by cell surface receptors that regulate lipopolysaccharide. TNF-*α* activated antiapoptotic and pro-inflammatory signaling. PI3K pathway is involved in regulating the growth, proliferation, recruitment, activation, and survival of leukocytes, playing a role in the pathogenesis of inflammation, tumors, metabolism, and other diseases [35, 36]. Serine/threonine-protein kinase (AKT), also known as protein kinase B (PKB), is a 57kD serine/threonine-protein kinase encoded by the proto-oncogene c-AKT, which is a direct target protein of PI3K. Activated AKT exerts regulatory effects on cell survival, growth, and proliferation. Nuclear factor-*κ*B (NF-*κ*B) is a transcription factor that plays a key regulatory role in gene expression and is involved in a variety of biological processes including immune response, inflammatory response, apoptosis, and tumorigenesis. NF-*κ*B can be activated by a variety of factors including inflammatory cytokines, mitogens, oxidative stress, infection, and microbial products. In the resting state, NF-*κ*B is present in the cytoplasm in an inactive form and binds to I*κ*B in the cytoplasm. Upon stimulation, I*κ*B-*α* is phosphorylated by a kinase (IKK), and the phosphorylated I*κ*B-*α* is ubiquitinated and degraded, leading to NF-*κ*B nuclear translocation and activation of transcription of target genes [37]. The activation of PI3K/AKT pathway enhances the phosphorylation and degradation of NF-*κ*B repressor protein I*κ*B, which activates NF-*κ*B and binds to specific intracellular DNA sequences to exert transcriptional and regulatory effects [38]. Also, the expression of the NF-*κ*B subunit increases PI3K activity and promotes AKT activation, increasing AKT expression at transcriptional levels [39]. As a regulator of the inflammatory response and a key effector molecule of the PI3K/AKT pathway, the transcription factor NF-*κ*B plays an important role in intestinal inflammation in UC. The PI3K/AKT/NF-*κ*B pathway is an important pathway that regulates cytokines, inflammatory responses, cell proliferation, differentiation, and death signals.

Intestinal inflammation and disruption of the intestinal epithelial barrier are the two main etiological factors triggering UC. There are complex interactions and cellular communication between different pro-inflammatory cytokines/chemokines, oxidative mediators, inflammatory cells, and immune cells during the progression of UC development. Inflammatory factors, immune cells, and microbial communities combine to form a pro-inflammatory microenvironment in the gut, which causes UC. Therefore, normalizing the abnormal inflammatory microenvironment of UC may be a novel effective treatment strategy [40]. Recently, several cytokines have been shown to affect the structure and function of the intestinal mucosal barrier, especially pro-inflammatory cytokines (e.g., IL-1*β*, IL-6, TNF-*α*, and IFN-*γ*) that play an important role in intestinal mucosal tight junctions and immune response. Apoptosis is an active form of programmed cell death under certain physiological or pathological conditions, controlled by apoptosis-related genes after cells receive stimulus signals. In the development of UC, accelerated apoptosis of colonic epithelial cells is an important cause of persistent inflammation. Studies have confirmed that apoptosis is one of the important mechanisms affecting intestinal mucosal injury and immune disorders in UC patients. It is mainly manifested by accelerated apoptosis of epithelial cells and delayed apoptosis of inflammatory cells, of which the coexistence is an important reason for the persistent inflammation. Intestinal epithelial cells are an important component of the intestinal mucosal barrier and rarely undergo apoptosis under normal conditions. To maintain the balance and basic function of the barrier, epithelial cells often participate in the apoptotic process through the action of various damage factors such as TNBS [41, 42]. Abnormal apoptosis of intestinal epithelial cells is a hallmark of TNBS-induced colitis. It disrupts the integrity and barrier function of the intestinal mucosa and leads to other changes associated with colitis [43]. Bcl-2 and Bax are important factors that regulate apoptosis. Bcl-2 can inhibit apoptosis, while Bax, a homolog of Bcl-2, is an apoptosis-inducing gene. They are also apoptotic factors associated with the PI3K/AKT/NF-*κ*B signaling pathway. Numerous studies on tumors have shown that PI3K signaling plays an important role in promoting tumor cell growth and antiapoptosis and in helping tumor cells gain immortality [44]. However, in cells other than tumor cells, PI3K signaling appears to play the opposite role, promoting apoptosis to maintain a steady state [45]. In colonic epithelial cells, apoptosis is induced by cytokine stimulation such as TNF-*α* and IFN-*γ*, and this process is mediated through the PI3K/AKT pathway. Abnormal activation of the NF-*κ*B signaling pathway can also affect apoptosis and cell cycle regulation. The PI3K/AKT/NF-*κ*B signaling pathway is a key signal that regulates apoptosis in intestinal epithelial cells, and activation of the pathway accelerates apoptosis in intestinal epithelial cells while promoting the persistence of a state of inflammation. Therefore, reducing the intestinal inflammatory response, promoting mucosal healing, and maintaining colonic barrier function are the therapeutic goals of UC.

In the experimental validation, we found that the expression of inflammatory factors TNF-*α*, IL-1*β*, and IL-6 in the colon was significantly elevated in UC model rats induced by TNBS, apoptosis of colonic epithelial cells was increased, Bax protein expression was increased, and Bcl-2 protein expression was decreased, while the above effects were significantly reversed after RBP treatment. RBP also decreased the expression of PI3K, AKT, and NF-*κ*B mRNA and protein in the colonic tissue of UC rats. These results suggest that RBP can ameliorate intestinal inflammation, reduce apoptosis of colonic epithelial cells, and maintain intestinal homeostasis and intestinal mucosal barrier by inhibiting the activation of PI3K/AKT/NF-*κ*B signaling pathway; reducing the release of inflammatory factors such as TNF-*α*, IL-1*β*, and IL-6; and regulating the expression of apoptosis-related factors Bax and Bcl-2, thereby achieving the treatment of UC.

## 5. Conclusions

**Figure 11:**
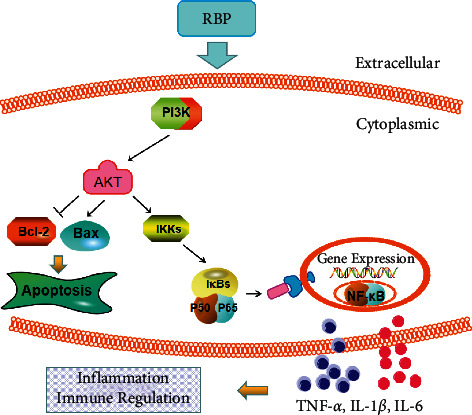
The mechanism schematic diagram of RBP. The arrows (⟶) represent the activating effect and the T-arrows (⊣) represent the inhibiting effect.

In summary, this study tackles the molecular mechanism of RBP in treating UC based on network pharmacology and experiment validation. Our results revealed that the anti-UC property of RBP may be achieved by inhibiting PI3K/AKT/NF-*κ*B signaling pathway. This research approach may serve as a practical strategy to facilitate pharmacological studies of TCM on complex diseases for relevant pathways and target screening. The mechanism schematic diagram of RBP is shown in Figure 11.

## Figures and Tables

**Table 1:** Polymerase chain reaction primers' gene sequences.

Target gene	Primer sequence	Product length in bp
PI3K	Forward: GCTGTTGATAGACCACCGCTTCC	89
	Reverse: TGCCCTGTTCCTCTGCCTTCC	

AKT	Forward: GCGGTCGATGCACTCCAGAAC	186
	Reverse: CACAGCCCGAAGTCCGTTA	

NF-*κ*B	Forward: GGGATGGCTTCTATGAGGCTGAAC	99
	Reverse: CTTGCTCCAGGTCTCGCTTCTTC	

GAPDH	Forward: GACATGCCGCCTGGAGAAAC	92
	Reverse: AGCCCAGGATGCCCTTTAGT	

## Data Availability

The datasets used and/or analyzed during the current study can be obtained from the corresponding author upon reasonable request.

## Abbreviations

ANOVA: Analysis of variance
ARA: Arachidonic acid
Bax: B-cell lymphoma-2-associated X
BCA: Bicinchoninic acid
Bcl-2: B-cell lymphoma 2
BSA: Bovine serum albumin
DAB: Diaminobenzidine
DNA: Deoxyribonucleic acid
ELISA: Enzyme-linked immunosorbent assay
GO: Gene ontology
HE: Hematoxylin-eosin
IBD: Inflammatory bowel diseases
IHC: Immunohistochemistry
IL-1*β*: Interleukin-1 beta
IL-6: Interleukin-6
ILs: Interleukin family
KEGG: Kyoto encyclopedia of genes and genomes
MPO: Myeloperoxidase
mRNA: Messenger RNA
NF-*κ*B: Nuclear factor-*κ*B
PBS: Phosphate-buffered saline
PCR: Polymerase chain reaction
PGs: Prostaglandins
PI3K: Phosphatidylinositol-3-kinase
PKB: Protein kinase B
RBP: Renshen baidu powder
RNA: Ribonucleic acid
RNS: Reactive nitrogen species
ROS: Reactive oxygen species
RT-QPCR: Real-time quantitative polymerase chain reaction
SASP: Sulfasalazine
SD: Sprague-Dawley
SMILES: Simplified molecular-input line-entry specification
SPF: Specific-pathogen-free
TCM: Traditional Chinese medicine
TGF-*β*: Transforming growth factor-*β*
TIC: Total ion current
TNBS: 2,4,6-trinitro-benzenesulfonicacid
TNF-*α*: Tumor necrosis factor-*α*
TUNEL: TdT-mediated dUTP Nick End Labeling
UC: Ulcerative colitis
UHPLC-Q-Orbitrap-HRMS: Ultrahigh-performance liquid chromatography quadrupole orbitrap high-resolution mass spectrometry.

## Supplementary Materials

Supplementary Materials Supplementary Materials. Supplementary File 1. Composition of Renshen Baidu Powder; Supplementary File 2. Manufacturers and product numbers of the reagents used in this study; Supplementary File 3. Conditions of the mass spectrometer; Supplementary File 4. Chemical components of RBP identified by UHPLC-Q-Orbitrap-HRMS.
