# Supplementary material for: Renshen Baidu Powder Attenuated Intestinal Inflammation and Apoptosis in Ulcerative Colitis Rats through the Inhibition of PI3K/AKT/NF-κB Signaling Pathway
Source: Evid Based Complement Alternat Med. 2022 Jul 30;2022:5234025. doi: 10.1155/2022/5234025 (PMC9356782; doi:10.1155/2022/5234025)
Supplement: Supplementary Materials — Supplementary File 1. Composition of Renshen Baidu Powder; Supplementary File 2. Manufacturers and product numbers of the reagents used in this study; Supplementary File 3. Conditions of the mass spectrometer; Supplementary File 4. Chemical components of RBP identified by UHPLC-Q-Orbitrap-HRMS. [file 5234025.f1.docx]

**Supplementary** **File 1**

**Composition of Renshen Baidu Powder**

| **Chinese name** | **Botanical plant name** | **Quantity in g** |
| --- | --- | --- |
| Chai Hu | *Bupleurum chinense* DC. | 12 |
| Chuan Xiong | *Ligusticum Chuanxiong* Hort. | 9 |
| Ren Shen | *Panax ginseng* C.A.Mey. | 6 |
| Qian Hu | *Angelica decursiva* (Miq.) Franch. & Sav. | 6 |
| Gan Cao | *Glycyrrhiza glabra* L. | 6 |
| Jie Geng | *Platycodon grandiflorus* (Jacq.) A.DC. | 6 |
| Qiang Huo | *Notopterygium incisum* K.C.Ting ex H.T.Chang | 6 |
| Du Huo | *Angelica dahurica* (Hoffm.) Benth. & Hook.f. ex Franch. & Sav. | 6 |
| Fu Ling | *Smilax glabra* Roxb. | 6 |
| Zhi Ke | *Citrus × aurantium* L. | 6 |
| Bo He | *Mentha canadensis* L. | 3 |
| Sheng Jiang | *Zingiber officinale* Roscoe | 3 |

**Supplementary** **File 2**

**Manufacturers and product numbers of the reagents used in this study**

2,4,6-trinitro-Benzenesulfonicacid (TNBS) (P2297) was purchased from Sigma Group (Shanghai, China). BCA protein concentration determination kit (AR0146), Rat TNF-α ELISA Kit (EK0526), Rat IL-6 ELISA Kit (EK0412), Rat IL-1β ELISA Kit (EK0393), and Apoptosis two-step assay kit (MK1014) were purchased from Boster Biological Technology Co., Ltd (Wuhan, China). RIPA lysis buffer (R0020) was purchased from Solarbio Co., Ltd (Beijing, China). Anti-fluorescence quenching sealer (G1401) provided by Servicebio (Wuhan, China). Animal Total RNA Isolation Kit (NO.RE-03014) was purchased from FOREGENE Co., Ltd (Chengdu, China). 5×All-In-One MasterMix (with AccuRT Genomic DNA Removal kit) and EvaGreen Express 2×qPCR MasterMix-No Dye were purchased from Applied Biological Materials Inc. (Canada). Bcl-2 antibody (GTX100064) was procured from GeneTex, Inc. (Alton Pkwy Irvine, CA, USA). NF-κB p65 antibody (8242) was purchased from Cell Signaling Technology Inc. (Boston, USA). Anti-Bax antibody (ab32503), anti-PI3Kinase antibody (ab180967), and anti-AKT antibody (ab179463) were purchased from Abcam (Cambridge, UK).

**Supplementary File 3**

**Conditions of the mass spectrometer**

For the UHPLC-Q-Orbitrap-HRMS analysis, mass spectrometric detection was carried out and positive ionization mode was used. The conditions of mass spectrometer were set as follows: full mass: 70,000, dd-MS2: 17,500, scan range: 150.0-2000.0m/z, spray voltage: 3.8 kV (positive), Capillary Temperature: 300℃, respectively. The HPLC analysis was performed with a Welch Ultimate column (AQ-C18, Maryland, California, United States) with a column temperature of 35℃. The linear-gradient mobile phase consisted of mobile phase A (0.1% formic acid in water) and mobile phase B (pure methanol). The flow phase gradient was set as follows: 0-1 min, 98% A, 2% B; 1-5 min, 80% A, 20% B; 5-10 min, 50% A, 50% B; 10-15 min, 20% A, 80% B; 15-20 min, 5% A, 95% B; 20-25 min, 5% A, 95% B; 25-26 min, 98% A, 2% B; 26-30 min, 98% A, 2% B;), with a 0.3 mL/min flow rate.

**Supplementary File 4**

**Chemical components of RBP identified by UHPLC-Q-Orbitrap-HRMS.**

| No. | Compound name | Formula | Molecular Weight | RT(min) | Adducts |
| --- | --- | --- | --- | --- | --- |
| 1 | Nicotinic acid | C6H5NO2 | 123.0323 | 2.59 | [M+H]^+^ |
| 2 | Adenosine 5'-monophosphate | C10H14N5O7P | 347.0629 | 3.05 | [M+H]^+^ |
| 3 | Phloroglucinol | C6H6O3 | 126.0320 | 3.65 | [M+H]^+^ |
| 4 | Adenosine | C10H13N5O4 | 267.0965 | 4.82 | [M+H]^+^ |
| 5 | Guanine | C5H5N5O | 134.0229 | 5.49 | [M+NH]^+^ |
| 6 | Indole-3-acrylic acid | C11H9NO2 | 187.0632 | 7.46 | [M+NH]^+^ |
| 7 | Caprolactam | C6H11NO | 113.0844 | 8.56 | [M+H]^+^ |
| 8 | Fraxetin | C10H8O5 | 208.0370 | 9.82 | [M+H]^+^ |
| 9 | cis-Resveratrol | C14H12O3 | 228.0782 | 10.94 | [M+H]^+^ |
| 10 | Scopoletin | C10H8O4 | 192.0420 | 11.60 | [M+H]^+^ |
| 11 | Naringenin | C15H12O5 | 272.0680 | 11.79 | [M+H]^+^ |
| 12 | Columbianetin | C14 H14O4 | 246.0886 | 12.68 | [M+H]^+^ |
| 13 | Isorhamnetin | C16H12O7 | 316.0579 | 13.70 | [M+H]^+^ |
| 14 | α-Lapachone | C15H14O3 | 242.0941 | 14.04 | [M+H]^+^ |
| 15 | 1,3-Dihydroxy-1-(7-methoxy-2-oxo-2H-chromen-6-yl)-3-methyl-2-butanyl 3-methylbutanoate | C20H26O7 | 378.1673 | 14.94 | [M+H]^+^ |
| 16 | Methoxsalen | C12H8O4 | 216.0421 | 15.08 | [M+H]^+^ |
| 17 | Sinapinic acid | C11H12O5 | 206.0578 | 15.53 | [M+H]^+^ |
| 18 | Diosmetin | C16H12O6 | 300.0631 | 15.98 | [M+H]^+^ |
| 19 | 7-Hydroxycoumarine | C9H6O3 | 162.0315 | 17.17 | [M+H]^+^ |
| 20 | 9-(Acetyloxy)-8,8-dimethyl-2-oxo-9,10-dihydro-2H,8H-pyrano[2,3-f]chromen-10-yl 2-methyl-2-butenoate | C21H22O7 | 408.1179 | 17.32 | [M+H]^+^ |
| 21 | 1-[2-(1,3-Benzodioxol-5-yl)-3-methyl-1-benzofuran-5-yl]-1,2-propanediol | C19H18O5 | 308.1046 | 18.67 | [M+H]^+^ |
| 22 | 9S,13R-12-Oxophytodienoic acid | C18H28O3 | 292.2037 | 18.82 | [M+H]^+^ |
| 23 | (1R,9S)-5-[(E)-2-(4-Chlorophenyl)vinyl]-11-(4-methylbenzoyl)-7,11-diazatricyclo[7.3.1.02,7]trideca-2,4- | C27H25ClN2O2 | 466.1393 | 19.34 | [M+H]^+^ |
| 24 | Ostruthin | C19H22O3 | 298.1564 | 19.80 | [M+H]^+^ |
| 25 | 18-β-Glycyrrhetinic acid | C30H46O4 | 470.339 | 21.15 | [M+H]^+^ |
| 26 | Hexadecanamide | C16H33NO | 255.2559 | 21.40 | [M+H]^+^ |
| 27 | Stearamide | C18H37NO | 283.2870 | 22.67 | [M+H]^+^ |
| 28 | 5,5'-[(6Z)-6-Tetradecene-1,14-diyl]bis(1,3-benzenediol) | C26H36O4 | 390.2763 | 23.32 | [M+H]^+^ |
| 29 | Docosanamide | C22H45NO | 339.3496 | 24.62 | [M+H]^+^ |
| 30 | Arachidic acid | C20H40O2 | 312.30281 | 25.96 | [M+H]^+^ |
